# Designing a Smart Bath Assistive Device Based on Measuring Inner Water Temperature for Bathing Temperature Monitoring

**DOI:** 10.3390/s20082405

**Published:** 2020-04-23

**Authors:** Qun Wei, So-Myoung Kang, Jae Ho Lee

**Affiliations:** 1 Department of Biomedical Engineering, School of Medicine, Keimyung University, Daegu 999007, Korea; weiqun@kmu.ac.kr; 2 B2B Smart Solution Team, LG U+ Inc., Seoul 999007, Korea; somyoung@lguplus.co.kr; 3 Department of Anatomy, School of Medicine, Keimyung University, Daegu 999007, Korea

**Keywords:** bath, water temperature measurement, water depth, IoT, curve fitting, compensation algorithm

## Abstract

Today, taking a bath is not only a means to keep clean, but also to reduce fatigue and stress. However, taking a bath with hot water for a long time can also be dangerous, leading to scalding or even a heart attack. To prevent these risks, several studies based on measuring bio-signals have been conducted, but due to high prices, difficulty of use, and restricted functions, these studies’ recommendations cannot be easily adopted by the public. Therefore, developing accurate methods to measure bathing temperature and bathing time should be the most direct approach to solve these problems. In this study, a smart bath assistive device based on an inner water temperature measurement function is proposed. Prior to development of the device, a bathing environment was emulated with six temperature sensors affixed to different depths to find the optimal depth for measuring bathing temperature. According to the measurement results, the device was designed in a mushroom shape with the cap part floating on the water’s surface and housing the electronic components, and temperature sensors within the stem part were immersed in the water approximately 5 cm below the surface to measure the inner water temperature. Due to the low-power consuming Advanced RISC Machine (ARM) processor and waterproof design, the device is able to float in hot water and monitor the bathing temperature variation over a long period of time. The device was compared alongside a commercial analog bathing thermometer to verify the performance of temperature measurements. In addition, a compensation algorithm was developed and programmed into the device to improve the accuracy of measurements. Processed data is transmitted by Bluetooth to a dedicated Android app for data display and storage. The final results show that the proposed device is highly accurate and stable for monitoring bathing temperature.

## 1. Introduction

Bathing not only maintains the cleanliness of the body, but also relieves fatigue, helps blood circulation and promotes metabolism [1]. For infants, taking a bath two to three times a week is recommended to prevent not yet fully developed sweat glands from clogging due to excessive sweating, which trap perspiration beneath the skin resulting in prickly heat, heat rash, or miliaria [2,3]. Also, as is widely known, taking a bath is effective for treating injuries, promoting good health, and preventing diseases [1,4]. Therefore, it is a favored option for most sick and elderly people, who cannot participate in regular physical activities, and can be easily done at home [5].

However, taking a bath at excessive water temperatures can lead to more harm than good, with unsafe water temperatures leading to shock and scalding, particularly among infants and elderly people. Because infants scald or burn easily due to their sensitive skin (i.e., infants have a 30% thinner stratum corneum and a 20% thinner epidermis) and are more easily affected by heat, the water temperature should be maintained at around 37~38 °C [6,7]. An 8-year retrospective review of patients admitted to Stoke Mandeville Hospital (Aylesbury, UK) due to burns sustained by hot baths or showers was undertaken in one study. Fifty-seven patients of all ages were identified and stratified into pediatric (<16 years) and adult groups. In the pediatric group, children were predominantly under three years of age (83%), sustaining most frequently only superficial burns (41%) over areas of less than 10% of total body surface area (72%). Also, parents’ supervision was inadequate in 85% of cases [8]. For elderly people, a half-full bath around 38~40 °C and for 20~30 min is suggested [9]. When the water temperature is higher than 41 °C, relaxation and contraction of blood vessels becomes more rapid, and it could aggravate angina and cardiovascular disease, as well as lead to blood pressure-related problems and pulse fluctuations. These physiological changes often result in syncope which can prove fatal in a bath [10,11,12,13]. Several studies with methods for preventing these risks have been presented, such as: monitoring and evaluating heart rates and peripheral blood flow during bathing; a system for measuring electrocardiogram (ECG) during bathing; and a variety of ubiquitous health monitoring systems to sense human bio-signals using sensors placed inside the bathtub when bathing at home [14,15,16]. However, both of these studies only focused on measuring bio-signals which led to very complex design structures, necessitating the integration of very expensive sensors and other components made from bio-compatible materials. Difficulty of use, restricted functions, and the high cost of developing these studies’ recommendations means they cannot be easily adopted by the public. Since water temperature is the most direct cause of bathing-related accidents, developing accurate methods to measure bathing temperature and bathing time, with real-time alerts to a smart phone, should be the simplest solution to overcoming the previous studies’ limitations.

Recently, common commercial bathing thermometers have been developed based on analog or digital temperature measurement techniques. Analog bathing thermometers, i.e., mercury thermometers, are the most popular approach to measuring water temperature due to their ease of use and high accuracy. However, the small scale of the thermometer can be hard to read, and the expansion variation of mercury is slow. In addition, the measured value cannot be observed and recorded expediently while the analog thermometer is in use. More importantly, mercury thermometers break easily, with broken glass and mercury’s high toxicity causing great harm. Digital thermometers are based on semi-conductor technology and are more commonly used to safely and accurately measure water temperature. The maximum error value of the temperature measurement is less than 0.3 °C, and they have fast calibration and response times. Also, the digital design and display allows for easier reading and recording of measurements.

According to the principles of heat transfer, heat dissipates more quickly at the water’s surface than under the surface, so different depths have different temperatures [17,18]. Also, research on absorption and attenuation of visible and near-infrared light in water that is temperature dependent has shown that water temperature dropped as depth increased [19]. Therefore, the water temperature measured by existing commercial bathing thermometers cannot determine exact bathing temperatures because they only measure the surface temperature. To overcome this deficiency, an IoT-based smart bath assistive device with an inner water temperature measurement function is proposed. Prior to development of the device, a bathing environment was emulated with a large glass water tank and six high accuracy temperature sensors affixed to different depths to find the relationship between depth and temperature variation (Section 2.2). According to the measurement results, the device was designed in a mushroom shape with the cap part floating on the water’s surface, which stores the electronic components for the system control, and a stem part immersed in the water at around 5 cm below the surface, which houses a monolithic complementary metal-oxide-semiconductor (CMOS) integrated circuit (IC) integrated temperature sensor to measure inner water temperature. A low-power consuming ARM processor with high performance and a long-distance Bluetooth 4.0 module were implemented in the device for system control, data processing, and wireless communication. Because the device was manufactured with waterproof design and a large capacitance rechargeable battery, the device is able to float in hot water to monitor bathing temperature for a long duration of time. The prototype device was compared alongside a current commercial bath thermometer to verify the performance of temperature measurement. In addition, a compensation algorithm was developed and programmed into the device to improve the accuracy of measurements. The processed data is transmitted by Bluetooth to a dedicated Android application for data display and storage.

## 2. Methods

### 2.1. Basic Idea of the Proposed Smart Bath Assistive Device

**Figure 1:**
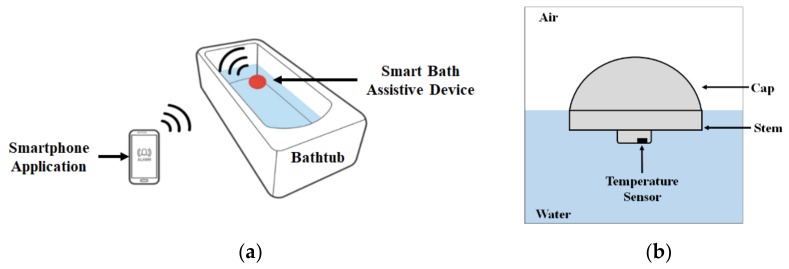
Basic idea of the proposed smart bath assistive device to monitor inner water conditions in the bathtub: **(a)** operating principle of the proposed system to monitor the bathing temperature; **(b)** design of the smart bath assistive device.

Figure 1a shows the basic idea of the proposed Internet of Things (IoT)-based smart bath-assistive device, represented as a red dot that can float alone in water to monitor bathing temperature. With a high-accuracy temperature sensor, wireless communication, and alarm function, the proposed device was developed to measure the bathing temperature and to send real-time alerts to a smartphone automatically. As mentioned, the surface heat of hot water dissipates more quickly on the surface than underwater wherein different depths have different temperatures in the bathtub. The mushroom shape of the proposed device, shown in Figure 1b includes a large cap area that allows the device to float in water unassisted, and a stem part that contains the temperature sensor to be immersed in water to measure the inner water temperature.

### 2.2. Observing the Relationship between Water Temperature and Depth

Convection is heat transfer by mass motion of a fluid such as air or water when the heated fluid is caused to move away from the source of heat, carrying energy with it. Hot water is less dense than cold water, causing it to rise and create convection currents which transport energy. Cooler, denser water descends, and warmer water rises near the surface. As was surmised, water temperatures below the surface of the bathtub were higher than water at the surface. However, given convection dynamics, deeper bath water is colder, so it had to be ascertained prior to designing the device which depth (how far below the surface, and how high above deeper, colder bath water) is optimal for measuring inner water temperature. 

**Figure 2:**
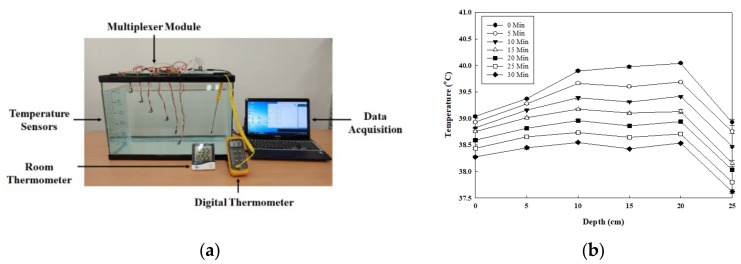
Experiment for observing the relationship between water temperature and depth: **(a)** picture of experiment setup; **(b)** result of the relationship between water temperature and depth over time.

Bathtub depths can vary: a European style bathtub has a depth of 45 cm, and a Japanese (or Greek) style bathtub has a depth of 55 cm. Standard bathtubs have depths between 35 and 44 cm, and a surface area of about 155 × 80 cm, which can hold between 95 and 170 L of water. According to the heat transfer rate equation for the water tank:

(1) $$Q=h\;A\left(T_{Ambient\_Temperature}-T_{Tank\_Water\_Temperature}\right)$$

h is heat transfer coefficient, A is surface area where the heat transfer takes place. Then, larger surface areas cause the overall heat transfer rate is larger than smaller surface areas. Also, natural convection occurs when there are hot and cold regions of water in the water tank, not generated by any external source and not depending on the volume of the water tank. Both of these theories meaning the speed of heat dissipation of the average bathtub is faster than that of the glass water tank [20]. However, this research focuses on finding the relationship between water temperature variation and difference depth only. Therefore, a 60 × 30 × 45 cm glass water tank with the same depth of a standard bathtub was filled up with 63 L of 40 °C water that is considered to be one of the most comfortable bathing temperatures to emulate the bathing environment, as shown in Figure 2a [10]. Six semi-conductor-based Si7021 (Silicon Labs, USA) temperature sensors were affixed individually at 5 cm intervals, starting from the surface down to a depth of 25 cm. The selected sensors have a very small maximum margin of measurement error, about ±0.3 °C at a 1 Hz sampling rate, which contributes to measuring the water temperature accurately. Also, this sensor is a monolithic CMOS IC integrated sensor. The 3 × 3 mm dual-flat no-leads (DFN) pack includes an analog-to-digital converter (ADC), signal processing, calibration data, and an inter-integrated circuit (I2C) interface, which make it easier to connect with the microcontroller unit (MCU) for system design — easier than dedicated water measurement sensors such as CS225 (Campbell Scientific, USA), TPJ 10K, and NTC10K (Capetti Elettronica, Italy). In addition, because of the monolithic CMOS sensor structure’s design, the sensor IC has low drift and hysteresis, and excellent long-term stability [21]. Before affixing the sensors in the water tank, all the sensors were coated with epoxy to make them waterproof, and the accuracy of the measurements was calibrated and compensated for via programing. The six sensors were connected to an 8-channel multiplexer to control the sensors and acquire the data simultaneously. Also, a TES 1300 thermometer (TES, Taiwan), capable of measuring temperatures in the range of –50 °C to +199.9 °C with an accuracy of ±0.03% rdg, was affixed at a depth of 20 cm to observe the overall temperature. After the sensor initialization, the experiment began and was conducted for 30 min at 25 °C room temperature, and data measured from all the sensors were recorded every 5 min. The experiment results are shown in Figure 2b: the water temperature increased from the surface to the 20 cm depth and then decreased dramatically at the 25 cm depth, which was lower than the surface temperature and became increasingly lower as time went by. Also, measured temperatures from 10 to 20 cm depths fluctuated significantly, and the lowest value from 5 to 20 cm depths can be observed at the 15 cm depth for the last measurement (30 min). Clearly, the water temperature at the 5 cm depth shows the most stable variation as time went by, more so than at other depths in the whole experiment. Therefore, the 5cm depth position was selected as the point from which to measure the water temperature due to its linear and stable variation, as well as for practical design considerations for the mushroom-shaped design.

**Figure 3:**
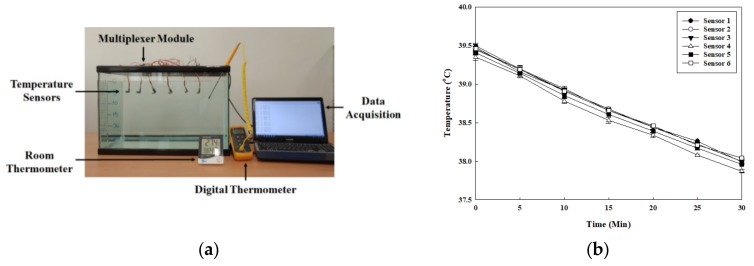
Experiment for observing water temperature variation in different locations at the same depth: **(a)** picture of the experiment; **(b)** results of water temperature variation in different locations at the same depth over time.

Considering the water flow in the bath environment the device cannot always stay in the same place in the bathtub. The water temperatures of different locations at the 5 cm depth was observed to study whether consistent temperature readings can be made when the device floats to different locations in the bathtub. As Figure 3a shows, the six sensors were distributed in different locations at the 5 cm depth to measure the water temperature for 30 min. The conditions of the bath environment were set up to match those of the previous experiment, and room temperature was also maintained as 25 °C. The experiment results show that the water temperatures measured from the six sensors were almost identical and decreased linearly over time, as seen in Figure 3b.

### 2.3. System Design of the Proposed Smart Bath Assistive Device

**Figure 4:**
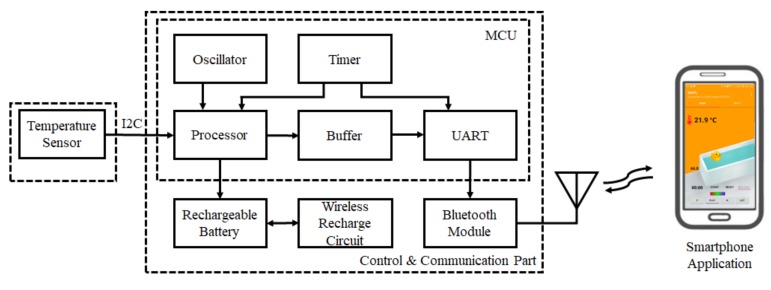
Block diagram of the proposed smart bath assistive device connected with a smartphone by Bluetooth.

Figure 4 shows the block diagram of the proposed smart bath assistive device that consists of three parts: the sensor part for measuring water temperature, the device control part with wireless communication, and the smartphone application for remote control of the device and data storage. The temperature sensor Si7021 used in the experiment of Section 2.2 was also used in the proposed device with a 1 Hz sampling rate for bathing temperature monitoring. The ARM Cortex-M4 core-based EFM32WG 32-bit microprocessor (Silicon Labs, USA) was selected as the main controller for the device’s control and data processing. This MCU provides a full digital system processor (DSP) instruction set and includes a hardware floating point unit (FPU) for faster computation performance. It also features up to 256 KB of flash memory, 32 KB of RAM, and central processing unit (CPU) speeds of up to 48 MHz, which are sufficient for implementing a compensation algorithm that processes the temperature automatically. With minimal energy consumption and intelligent peripherals, this MCU can efficiently control the device over a long lifespan. For the power supply, due to the nature of the device’s use, it’s not suitable to have a USB port to charge the battery. Therefore, a wireless charging circuit with wireless charging coils was designed for the device. 

For wireless communication between the device and smartphone, Bluetooth module BC127 (Blue Creation, USA) was selected. This module is highly flexible, with low-power consumption and a maximum data rate of 3 Mbps. In addition, an integrated antenna is ideal for easily adding high-quality audio and data communication. An Android OS-based smartphone app was developed to remotely control the proposed device, while the measured data is displayed and recorded for analysis via the app.

## 3. Experiment

### 3.1. Device Manufacture and Assembly

**Figure 5:**
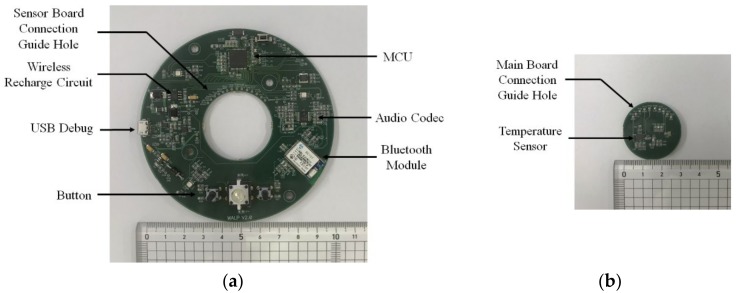
Front view of the manufactured PCBs of the smart bath assistive device: **(a)** main board; **(b)** sensor board.

Because of the proposed smart assistive bath device’s mushroom-shaped design, the printed circuit boards (PCBs) were manufactured as two separate parts: the main board with a larger surface area designated to mount the MCU, Bluetooth module, and other electronic elements are housed in the cap of the device; and a smaller circular board designated for the temperature sensor is housed in the bottom stem of the device, as shown in Figure 5a,b. For the main board, the Bluetooth module was mounted on the top of the PCB and kept away from the MCU to avoid tracking signals produced by the high-frequency oscillator of the MCU. Also, the antenna of the Bluetooth module is located on the edge of the PCB to avoid radiation pattern blockage caused by other electronics components. An audio codec is implemented to give safety alarms in real time associated with excesses in bath temperature and bathing time. Also, the audio codec can allow for communication between a caregiver and a user in the bath by hands-free Bluetooth technology. The wireless charging circuit was also designed on the top of the PCB and the receiver coil was attached to the back of the main PCB for direct connection to the transmission coil for the device’s docking and power transmission. Guide holes are designed on the same side of the two PCBs to connect easily to the main board. Both PCBs were manufactured with a four-layer structure, and all of the components’ sizes were from the 2012 package (2.0 × 1.25 mm) and were mounted on both sides of the PCB to minimize the PCB size.

**Figure 6:**
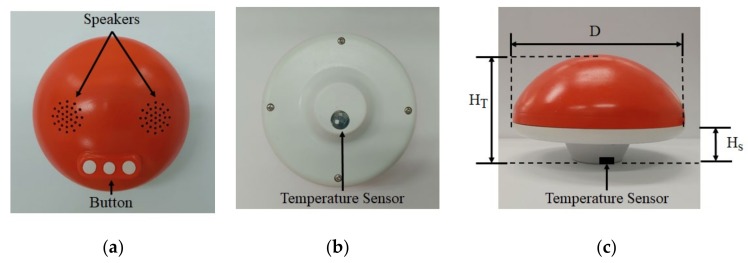
Pictures of assembled smart bath assistive device: **(a)** top view; **(b)** bottom view; and **(c)** lateral view.

Figure 6 shows the proposed smart bath assistive device assembled with manufactured mushroom-shaped shell. The shell is composed of a red cap and white stem fabricated by a 3D printer using high-impact strength acrylonitrile butadiene styrene copolymer (ABS) material. The ABS material has several advantages including high chemical resistance (making them safe to use), good electrical insulants, and, importantly, heat resistance that protects the device from deformation even when used in hot water with strong temperature variation [22]. Also, due to the ABS material being metal-free, it is able to avoid detuning effects on the antenna and unwanted signal path loss caused by the metal and metal coating housing material used to encapsulate the PCB. Figure 6a is the top view of the assembled device: two speakers for stereo sound and three buttons to control the device, all of which are waterproof. Figure 6b is the bottom view of the device: the temperature sensor was affixed to the bottom of the device and can be viewed clearly due to its thin and transparent acrylic cover. Four screws keep the cap and stem sealed together, with a rubber ring between cap and stem acting as an airtight and waterproof sealant. Figure 6c is the lateral view of the device showing its dimensions. The diameter (D) of the device is 12 cm and the total height of the device from the top to bottom of the device (HT) is 10 cm. The height of the stem part (HS) is 5 cm, which is immersed in the water allowing the temperature sensor to be 5 cm under the water.

### 3.2. Experiments for System Performance Test

#### 3.2.1. Temperature Sensor Calibration and Wireless Communication Performance Verification

Due to the temperature sensor and Bluetooth module being encased in the waterproof shell, a temperament measurement test in a temperature and humidity chamber (T2, YMRTC Co., Ltd.) for sensor calibration and a wireless communication performance test had to be conducted. After the chamber temperature was initialized at 40 °C, the device was placed in the center of the chamber and the chamber was then turned off in order to naturally bring down the temperature to 20 °C to imitate bathing temperature variation. Data measured by the device were transmitted to a laptop by Bluetooth and instantly recorded as raw data. However, the chamber temperature displayed on the chamber interface was measured by the chamber’s own sensor which is placed at the top of the chamber. Thus, the chamber temperature indicated was not suitable as a reference for observing the temperature near the device’s sensor [23]. Therefore, the K-type temperature sensor of the digital thermometer (TES-1300, TES Electrical Electronic Corp.) was attached near the device’s sensor. The measured data was also recorded as raw data by the digital thermometer to act as a comparative reference with the data measured by the smart bath assistive device.

#### 3.2.2. Experiment for Comparison of the Proposed Device with a Commercial Bath Thermometer

**Figure 7:**
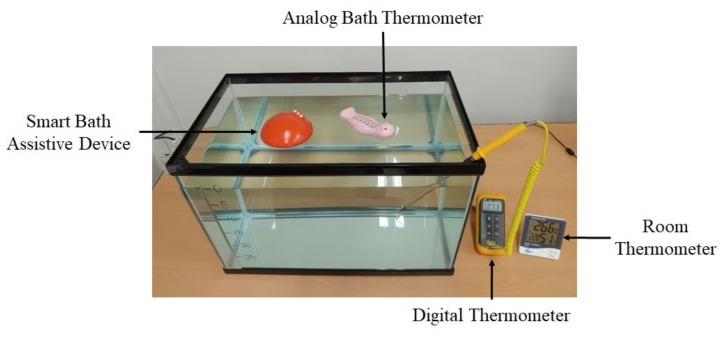
Experiment for comparison of the proposed system with a commercial analog bath thermometer.

As shown in Figure 7, the smart bath assistive device and an analog bath thermometer (Double Heart, Japan) were positioned to float in the water tank to compare their respective performances. The water tank was filled with 63L, 40 °C water (as done in the previous experiment) in order to observe the relationship between water temperature and depth. The experiment began at 40 °C and lasted for 30 min. The data measured by the device was recorded as raw data and the temperature values of the analog bath thermometer were recorded 10 times at one-minute intervals. In addition, a digital thermometer was set at a depth of 5 cm under the surface to observe temperature variation. The measured data were used as a reference to compare with the smart bath assistive device. Room temperature was also maintained at around 26 °C for the whole experiment.

## 4. Results and Discussion

**Figure 8:**
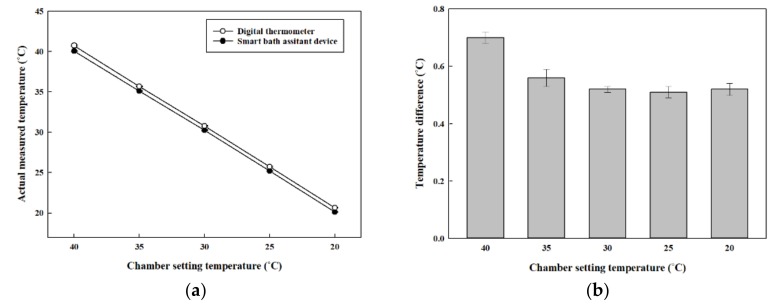
Results of the proposed smart bath assistive device in temperature measurement and wireless data communication performance test: **(a)** temperature variation; **(b)** temperature difference.

Figure 8 shows the experiment results of the proposed smart bath assistive device for temperature measurements and the wireless data communication performance test. The lines with black and white dots in Figure 8a represent the temperature measured by the proposed device and digital thermometer HTC-1, respectively. The x-axis and y-axis represent the chamber temperature versus the actual measured temperature. The temperature measured by the proposed device is lower than that of the digital thermometer. Figure 8b shows the gap between the two devices’ measurements starting around 0.7 °C and then gradually becoming smaller as the chamber temperature decreased. After the chamber temperature dropped to below 35 °C, the temperature difference between the two devices stabilized at around 0.5 °C until the chamber temperature reached the lowest temperature of 20 °C. As mentioned before, the device’s temperature sensor was enclosed within a waterproof shell, and even the cover of the sensor was made with a thermally sensitive material, meaning that the sensor was not able to measure the temperature directly. Therefore, the temperature measuring response speed of the proposed device is slower than that of the digital thermometer, causing the temperature measurement difference between the two devices, with the proposed device’s reading always a bit lower than the digital thermometer’s reading.

**Figure 9:**
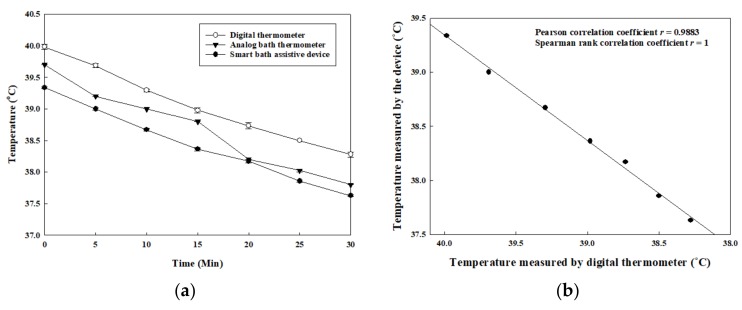
Graphs of the experiment results for comparison of proposed device, a commercial analog bath thermometer, and a digital thermometer for water temperature measurement: **(a)** water temperatures measured by the three devices; **(b)** Pearson and Spearman correlations of the proposed device and the digital thermometer.

Figure 9 shows the results of the second experiment: comparison of the water temperature measurement performance of the proposed device and the commercial device. Three temperature values were acquired by the proposed device, a commercial analog bath thermometer, and a digital thermometer as shown in Figure 9a. Although it was shown earlier that the inner bath-water temperature is higher than the temperature at the surface, the experiment results of the three devices show that the temperature measured by the analog device is a little higher than that of the proposed device. According to the results of the first experiment, the reason for this phenomenon is assumed to be due to the device’s sensor being enclosed within the stem, making the measured temperature lower than the actual temperature. In addition, the relationship between the device and the digital thermometer shows that: first, the digital thermometer’s measurement is higher than those of both the device and the analog bath thermometer, proving that the water temperature at a depth of 5 cm is again higher than at the surface; and second, the gap between the device and the digital thermometer remained constant from the experiment’s start to its finish, as can be clearly observed. Therefore, the relationship between the two values were studied by Pearson correlation and Spearman rank correlation in MATLAB, as shown in Figure 9b with the coefficient values of 0.9983 and 1, respectively, which proves that the two linear lines are almost exactly parallel. The difference between the two devices’ measurements was also close to 0.5 °C, which is similar to the results of the first experiment.

To compensate for the lower temperature value measured by the smart bath assistive device, a composition of the temperatures measured by the digital thermometer and by the device were studied from the experiment results, as represented by Equations (2) and (3), respectively. The temperature value acquired from the digital thermometer was assumed to consist of the actual temperature at the 5 cm depth and the error value of the digital thermometer. However, the temperature value acquired from the device not only consisted of the same temperature at the 5 cm depth and the error value of the sensor (Si7021), but also the error value that occurred due to the acrylic cover, which has to be taken into consideration. Furthermore, the margin of error of the digital thermometer in the range of 30~40 °C is approximately around ±0.1 °C, which is smaller than that of the device’s sensor at about ±0.2 °C. For these reasons, the main cause for the difference between the two devices’ measurements can be chalked up to the role of the acrylic cover and the error value of the proposed device’s sensor. In addition, the water temperature at the 5 cm depth measured by the digital thermometer can be used as the standard value to develop a compensation algorithm for improving accuracy of the device.

**Figure 10:**
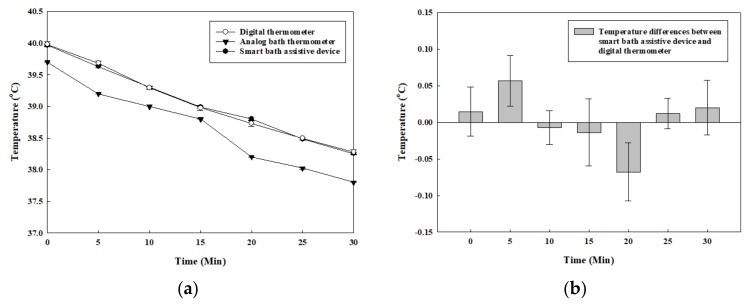
Graph with developed compensation algorithm for temperature measured by the proposed device: **(a)** water temperatures measured by the three devices; **(b)** differences in value between the proposed device (with compensation algorithm) and digital thermometer.

Therefore, the compensation algorithm based on the curve fitting method with a linear fitting model was suggested as Equation (4) [24]. Each coefficient for the equation was set with 95% confidence bounds. For evaluating the goodness of fit, the following values were observed: the sum of squares due to error (SSE) = 0.006552, R-square = 0.9972, adjusted R-square = 0.9958, and root mean square error (RMSE) = 0.04047. The data processed through the compensation algorithm can be seen in Figure 10. The compensation algorithm-processed temperature value of the device and the value of the digital thermometer are both higher than the analog thermometer by about 0.3 °C, as shown in Figure 10a, and the temperature difference between the proposed device and the digital thermometer is less than 0.03 °C, as shown in Figure 10b. Likewise, several studies have proposed a variety of design methods with complex systems to measure water temperature in baths. They have one thing in common: the temperature sensors were set at varying depths in the water to achieve high accuracy and high stability [25,26,27]. However, as this study shows, the proposed device has a small size, simple structure, and features the adaptable compensation algorithm that allows the device to measure the inner water temperature with high accuracy and stability for a long period of time. Although, there are several kinds of commercial wireless water temperature measuring devices such as ALA (Monnit, USA) and TMU (LinkThru, USA) for monitoring temperatures in water storage tanks, pools, and aquariums, these devices must be mounted on the inner wall surface of the water container and can only measure the temperature near the sensor—they cannot be moved to another place. In addition, the accuracy of these two devices are +/– 1 °C, which is two times lower than the proposed device’s accuracy of +/– 0.5 °C. Furthermore, as regards convenient usage and data acquisition, these commercial devices are at a disadvantage since they use a 900 MHz wireless communication frequency which necessitates connecting to a gateway first rather than communicating directly with a smartphone.

(2) $$T_{Digital\_Thermometer}=T_{5cm\_Depth}+T_{Error\_Digital\_Thermometer}$$

(3) $$T_{Proposed\_Device}=T_{5cm\_Depth}+T_{Error\_Acrylic}+T_{Error\_Sensor}$$

(4) $$T_{Actual\_Bath}=0.09102\;sin\left(T_{Proposed\_Device}-pi\right)+0.0189\;{\left(T_{Proposed\_Device}-10\right)}^{2}+23.83$$

## 5. Conclusions

In this paper, a smart bath assistive device measuring inner water temperature for monitoring bathing temperature was developed. Unlike current studies’ methods of measuring the water temperature at the surface of the bathwater with commercially available thermometers, the proposed device measures bathing temperature under the water at a depth of 5 cm due to the inner water’s temperature being higher than at the surface, which was shown by a basic experiment that observed the relationships between water temperatures and depths. The device was designed in a mushroom shape that consisted of a cap and a stem that stored the main PCB and the sensor PCB, respectively. The entire device was manufactured with a waterproof design allowing for the cap part to float in water, and the stem part to be immersed in the water to measure the inner water temperature. Two kinds of experiments were conducted for testing the performance of temperature measurement and for comparison of the proposed device with a commercial bath thermometer. According to the experiment results, a compensation algorithm was developed and programmed into the device to adjust the data measured by the smart bath assistive device. The processed temperature value was higher than the commercial analog bath thermometer by about 0.3 °C, which is similar to the basic experiment results. Also, the data acquired by the device exhibited greater linearity and stability than the analog thermometer, showing that the device has dependable performance in bathing temperature measurement. With a high-performance Bluetooth module, the processed data is transmitted to an Android OS-based smartphone app for display and storage. In addition, because of a low-power-consuming ARM architecture-based MCU for the system control and data processing, using two 3500 mAh rechargeable batteries, the system can work on one full charge for about 30 h.
